# Pregnancy History, Hypertension, and Cognitive Impairment in Postmenopausal Women

**DOI:** 10.1007/s11906-019-0997-9

**Published:** 2019-11-18

**Authors:** Kathleen B. Miller, Virginia M. Miller, Jill N. Barnes

**Affiliations:** 1 Department of Kinesiology, University of Wisconsin-Madison, Madison, WI USA; 2 Department of Surgery, Mayo Clinic, Medical Sci Bldg 421, 200 First St SW, Rochester, MN 55905 USA; 3 Department of Physiology and Biomedical Engineering, Mayo Clinic, Medical Sci Bldg 421, 200 First St SW, Rochester, MN 55905 USA

**Keywords:** Alzheimer’s disease, Gray matter, Menopause, Neurovascular control, Preeclampsia, White matter hyperintensities

## Abstract

**Purpose of Review:**

Risks for developing cardiovascular disease and cognitive decline increase with age. In women, these risks may be influenced by pregnancy history. This review provides an integrated evaluation of associations of pregnancy history with hypertension, brain atrophy, and cognitive decline in postmenopausal women.

**Recent Findings:**

Atrophy in the occipital lobes of the brain was evident in women who had current hypertension and a history of preeclampsia. Deficits in visual memory in women with a history of preeclampsia are consistent with these brain structural changes. The blood velocity response to chemical and sympathoexcitatory stimuli were altered in women with a history of preeclampsia linking impairments in cerebrovascular regulation to the structural and functional changes in the brain.

**Summary:**

Having a history of preeclampsia should require close monitoring of blood pressure and initiation of anti-hypertensive treatment in perimenopausal women. Mechanisms by which preeclampsia affects cerebrovascular structure and function require additional study.

## Introduction

Studies have repeatedly demonstrated an association between hypertension and Alzheimer’s disease and other dementias [1–3]. Sex difference in the incidence of hypertension between men and women has been reported for over 50 years [4, 5]; however, previous studies have not considered associations between hypertension and Alzheimer’s disease relative to sex, hormonal status, or other potential risk factors that might differ between men and women [6]. Young women have lower blood pressure and less prevalence of hypertension compared with young men [5]. Yet, the risk for developing hypertension increases in women at menopause. Along with menopause, there are female-specific conditions that may augment risk for both hypertension and cognitive decline. For example, the abrupt loss of ovarian hormones following bilateral oophorectomy before the age of 45 years increases the incidence of cardiovascular disease and neurological conditions [7••]. In addition to surgical menopause, pregnancy is considered a stress test for cardiovascular health which has implications for structural and functional changes in the brain. Hypertensive pregnancy disorders, such as preeclampsia, increase the risk for hypertension later in life by 3 to 4 times that of women who had a normotensive pregnancy [8, 9]. Importantly, many studies of women with a history of preeclampsia only evaluate women a few years postpartum and do not consider the combined effect of menopause and pregnancy history on cardiovascular and brain health. Thus, the purpose of this review is to provide an integrated evaluation of associations of pregnancy history, specifically a history of preeclampsia, with development of hypertension, brain atrophy, and cognitive decline in postmenopausal women.

## Hypertension, Cognitive Decline, and Alzheimer’s Disease

A 2016 scientific statement from the American Heart Association identified strong evidence for the detrimental impact of midlife hypertension on late-life cognitive function, thus highlighting the importance of considering hypertension as a major contributor to accelerated cognitive decline [10, 11••]. The concept that hypertension influences cognition was identified as early as the 1960s when it was discovered that pilots and air traffic controllers with hypertension had reduced psychomotor speed compared with controllers without hypertension [12]. In addition, a longitudinal study published in 1971 suggested “significant intellectual loss” over a 10-year period in hypertensive individuals that was not evident in age-matched normotensive individuals [13]. Since then, multiple other studies have provided evidence suggesting that individuals with hypertension, especially during midlife, have increased risk for cognitive decline, independent of stroke [14]. The mechanisms by which hypertension increases risk for cognitive decline are not fully understood. Neuroimaging studies in humans have identified structural brain changes normally associated with cognitive decline in hypertensive individuals such as reduced gray matter brain volume coupled with increased volume of white matter hyperintensities (WMH) [15, 16]. Importantly, brain atrophy in hypertensive individuals is evident in regions typically susceptible to Alzheimer’s disease pathology. In addition to structural brain changes, there are declines in function of the cerebral blood vessels in hypertensive individuals [17] including lowered cerebral blood flow [18], altered cerebral autoregulation [19], and impaired neurovascular coupling [17]. These changes link cerebrovascular function to increases in the brain’s susceptibility to elevated systemic blood pressure. Hypertensive individuals also demonstrate higher levels of Alzheimer’s disease related pathology compared to normotensive controls, such as reduced glucose metabolism [20], increased β-amyloid plaques [21], and neurofibrillary tangles [22], all which may be related to the aforementioned reductions in function of the cerebral blood vessels. It is possible that hypertension-induced impairments in brain structure or neurovascular dysfunction may precede development of Alzheimer’s disease pathology and occur years before there are observed reductions in cognitive dysfunction [23, 24]. Therefore, managing hypertension in midlife could have subsequent cognitive effects in later-life, but these ideas are difficult to study and require a longitudinal approach. Clinical trials aimed at controlling blood pressure in order to mitigate declines in cognitive function or reduce the risk of Alzheimer’s disease are ongoing with promising preliminary results [25, 26]. Although the Systolic Blood Pressure Intervention Trial (SPRINT) did not see a reduction in probable dementia with anti-hypertensive therapy, the study likely did not extend long enough to capture changes in cognition, as the median intervention lasted only 3.3 years. Furthermore, the SPRINT trial, among others, lacks information about how sex and hormonal status may impact the effects of hypertension on cognitive function. Thus, the cognitive consequences of sex-specific conditions of hypertension, such as hypertension during pregnancy or preeclampsia, are only beginning to be understood. Future studies on hypertensive women and specifically women with a history of hypertensive pregnancy disorders are warranted.

## Preeclampsia

Preeclampsia is defined as de novo hypertension and proteinuria or other evidence of significant end-organ damage after 20-week gestation [27]. The incidence of preeclampsia is approximately 2–8% worldwide, occurring in approximately 4% of pregnancies in the USA [28]. Preeclampsia increases fetal risk, as 20% of cases result in preterm birth which can augment risk for perinatal mortality and morbidity [29]. In addition, maternal risk is also increased, as preeclampsia can develop into eclampsia (seizure) and cause severe neurological damage including death [30]. Despite blood pressure returning to normotensive levels after delivery, women with a history of preeclampsia are at a higher risk for cardiovascular disease [31], including hypertension and ischemic heart disease, and renal disease [32]. In addition, and as highlighted in this review, there are long-term effects of preeclampsia on brain structure, cerebrovascular function, and cognition.

The pathophysiology of preeclampsia is unknown. Preeclampsia is associated with the failure of the spiral arteries of the decidua and myometrium to remodel early during pregnancy subsequently reducing blood flow to the placenta [33, 34] and increasing placental oxidative stress [35]. There is evidence to suggest that the placenta then secretes anti-angiogenic factors [36, 37], resulting in widespread maternal vascular dysfunction [38] and elevated sympathetic nervous system activity [39, 40]. Risk factors for developing preeclampsia include insulin resistance and diabetes [relative risk (RR) 3.7], chronic hypertension (RR 5.1), obesity (RR 2.8), chronic kidney disease (RR 1.8), lupus (RR 1.8), anti-phospholipid syndrome (RR 2.8), a family history of preeclampsia (2.9), and advanced maternal age (1.2) [41]. Multifetal pregnancy (RR 2.9) is also a significant risk factor for preeclampsia. In addition, a past history of preeclampsia increases the risk for preeclampsia for subsequent pregnancies 8-fold (RR 8.4) [42, 43]. Although many risk factors for preeclampsia suggest preexisting autonomic or autoimmune dysfunction, these are not obligatory for preeclampsia to develop. Thus, whether the aforementioned risk factors are causal, or pregnancy exacerbates a preexisting propensity for hypertension, is yet to be understood.

## Evaluating the Long-Term Effects of Preeclampsia

Many epidemiological studies have retrospectively addressed the effects of preeclampsia on maternal cardiovascular disease risk [9, 44], as well as the association between preeclampsia and risk for maternal cognitive decline [45••]. Few studies, however, have focused on the effect of hormonal changes on aging-related pathophysiology or integrated associations between vascular function and brain pathology because such studies require long-term follow-up and continuous funding across several generations of investigators. It is possible to leverage large clinical databases to evaluate women prospectively based on pregnancy history. These databases can then be used to examine longitudinal effects of hypertensive pregnancy or preeclampsia on cardiovascular and brain health. For example, the Rochester Epidemiology Project medical records linkage system was used to identify from a larger population-based cohort, age- and parity-matched women with and without histories of preeclampsia [46••]. These women were postmenopausal with the average age of 60 years and about 35 years past the incident pregnancy. The following sections of this review will highlight some of the findings in this cohort of postmenopausal women with different pregnancy histories.

## Preeclampsia and Cardiovascular Function

In the cohort of postmenopausal women identified as clinically asymptomatic for cardiovascular disease through the Rochester Epidemiology Project, the incidence of hypertension and use of anti-hypertensive medications were more frequent in women with a history of preeclampsia [46••]. Furthermore, upon computed tomographic (CT) imaging of their coronary arteries, women with a history of preeclampsia had significantly greater accumulation of calcium in the coronary arteries compared with women who had a history of a normotensive pregnancy [46••]. In addition, carotid intima-media thickness was greater in women with a history of preeclampsia compared to those with a history of a normotensive pregnancy, indicating perhaps a more generalized risk for cardiovascular disease [47••] and structural changes in large and small peripheral arteries.

In addition to the structural changes observed in the coronary and carotid arteries, it is possible that a history of preeclampsia may be associated with altered central hemodynamics. Altered central hemodynamics may not be obvious by evaluating standard clinical brachial blood pressures, as changes in the structure of the central elastic arteries may precede clinical changes in brachial blood pressure [48] and manifest as increased arterial stiffness or elevated central blood pressure. Importantly, increases in central blood pressure may be more influential than brachial blood pressure when evaluating the hemodynamic stress on target organs, especially considering the proximity of the target organs (such as the brain) to the heart [49]. Thus, even if brachial blood pressure is within a normative range, central blood pressure and arterial stiffness may be associated with vascular brain injury. In this context, fraction of WMH, a biomarker for cognitive decline [50, 51], associated with increasing central arterial blood pressure in postmenopausal women [52]. Women with preeclampsia have increased central arterial stiffness, which augments central blood pressure, during pregnancy [53, 54], and central arterial stiffness remains elevated during the months postpartum [55]. In women evaluated 35 years postpartum, there was an inverse relationship between central blood pressure and cognitive function scores in postmenopausal women with a history of preeclampsia but not in women with a history of a normotensive pregnancy (unpublished observations from our group). These observations suggest underlying vascular dysfunction in the large elastic arteries that may impact cerebrovascular function and ultimately cognition.

## Preeclampsia and Cerebrovascular Function

The mechanisms by which hypertension, or history of preeclampsia, influences the function of the cerebral blood vessels are beginning to be explored in preclinical studies. Associated changes in the structure and function of the central elastic arteries may affect the cerebral circulation, as the central elastic arteries are responsible for dampening large pulsatile forces from myocardial contraction that can translate to the brain’s microcirculation. Myogenic function of the cerebral arteries is essential for maintaining cerebral autoregulation. Cerebral autoregulation protects the brain against elevated blood pressure, which occurs during a preeclamptic pregnancy. For example, in animal models of preeclampsia, intracranial cerebral arteries [56] and hippocampal arterioles [57] do not remodel and have impaired myogenic tone [58] in response to pregnancy. The loss of autoregulation may result from dysregulation of ion channels in the cerebral parenchymal arterioles [59••]. A consequence of impaired cerebral autoregulation during a hypertensive condition such as preeclampsia is hyperperfusion of the brain, which may increase permeability of the blood-brain barrier and cause neuroinflammation (see [60•] for review). In this context, animal models of preeclampsia have demonstrated that hyperperfusion results in lasting damage to the cerebral vasculature and blood-brain barrier [56, 61].

In humans, cerebrovascular function can be assessed by measurable changes in cerebral blood flow in response to a stimulus (e.g., increased CO_2_). This change is defined as cerebrovascular reactivity (CVR) and is thought to reflect the function of the cerebral vessels (i.e., arteries, arterioles, capillaries) [62]. CVR is reduced in populations with cognitive impairment, supporting an association between CVR and cognitive decline [63–65]. Women with preeclampsia have lower CVR during pregnancy compared with women with a normotensive pregnancy [66, 67]. In the cohort of postmenopausal women with a history of preeclampsia, CVR was lower than in women with a history of a normotensive pregnancy even 35 years after the incident pregnancy [68••]. Reductions in CVR in women with a history of preeclampsia were associated with increased vascular activation [68••]. In addition, these postmenopausal women with a history of preeclampsia demonstrated an augmented cerebral blood velocity response to a sympathoexcitatory stimulus compared with women with a history of a normotensive pregnancy (unpublished observations from our group). Taken together, these results suggest that a history of having preeclampsia is associated with impaired neurovascular control. It is important to consider that it is unknown if differences in central blood pressure, vascular activation, and CVR may exist among women prior to pregnancy and, if so, might represent a unique predisposing phenotype for preeclampsia. Regardless, impaired neurovascular control may cause chronic hypoperfusion leading to brain structural changes associated with cognitive decline. Alternatively, impaired neurovascular control may directly affect cognitive function due to impairments in neurovascular coupling. Future studies should continue to utilize animals and humans to evaluate how preeclampsia contributes to impairments in cerebral blood vessels, which will ultimately help elucidate the link between preeclampsia, cerebrovascular dysfunction, and cognitive decline.

## Preeclampsia and Brain Structure

In addition to the evidence suggesting that a history of preeclampsia is associated with reduced cerebrovascular function, it is possible that preeclampsia also influences brain structure. Indeed, studies that have evaluated women less than 10 years postpartum suggest that a history of preeclampsia is associated with increased WMH volumes [69–71], typically associated with increased risk for accelerated cognitive decline [50, 51]. Few studies, however, have evaluated brain structure in postmenopausal women with a history of preeclampsia. One population-based study evaluated brain volume in women decades postpartum and found that women with a history of preeclampsia had reduced gray matter volumes compared with women with a history of a normotensive pregnancy [72], even after adjustment for other cardiovascular risk factors. Taken together, these findings suggest that a history of preeclampsia has an impact on both white matter and gray matter structures that is apparent 10+ years postpartum.

Having ongoing or current hypertension may also impact changes in gray matter volume depending on pregnancy history. In women with a history of preeclampsia and a current diagnosis of hypertension, gray matter volume was reduced compared with women with a history of preeclampsia who were normotensive and women with a history of a normotensive pregnancy [73••]. Using a voxel-based analysis, those women with a history of preeclampsia and current hypertension demonstrated gray matter atrophy in the occipital cortex compared with women with a history of preeclampsia who were normotensive [73••]. In addition, those with a history of preeclampsia and current hypertension had reduced gray matter volume in both prefrontal and sensorimotor cortices in both hemispheres compared with women who had a history of a normotensive pregnancy and were currently normotensive [73••]. These findings suggest that there are specific regions of the brain that may be more sensitive to injury by preeclampsia, and that brain atrophy may be exacerbated by current hypertension. The presence of WMH and evidence of brain atrophy are consistent with other biomarkers of accelerated brain aging and increased risk for cognitive decline. Future studies can identify how other neuroimaging biomarkers of cognitive decline are affected by pregnancy history.

## Preeclampsia and Cognitive Decline

Epidemiological evidence suggests that there is an association between a history of preeclampsia and cognitive decline [45••]; however, few studies have evaluated this idea prospectively. In a cohort of postmenopausal women with otherwise low cardiovascular risk, women with a history of preeclampsia demonstrated deficits in visual memory reflecting damage to the occipital region of the brain, consistent with findings regarding gray matter atrophy in the occipital lobe [74••]. In addition, there was a trend for women with a history of preeclampsia to be clinically diagnosed with mild cognitive impairment more frequently than women with a history of a normotensive pregnancy [74••]. These women with a history of preeclampsia also exhibited a more diffuse range of cognitive impairment [74••]. These findings 35 years postpartum extend results from studies of women with a history of preeclampsia less than 10 years postpartum suggesting a high incidence of cognitive complaints [75–77], as well as reduced motor speed, attention, and learning and memory performance [75, 77, 78]. The findings from this cohort of postmenopausal women add to the growing body of literature identifying the detrimental impact of hypertension on cognition later in life. Because preeclampsia is a hypertensive episode with potential lasting effects on the central vasculature, cerebral vasculature, and brain structure, future studies should further investigate the impact of preeclampsia on cognitive function. In addition, future studies should evaluate how pregnancy history effects cognitive function over the life span, including during times of hormonal flux during perimenopause and into postmenopause.

## Conclusions

**Fig. 1:**
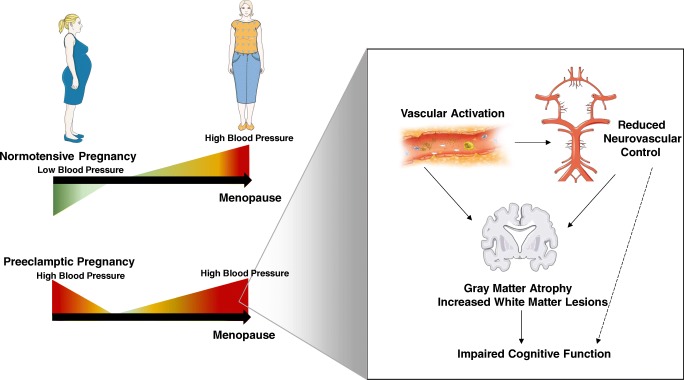
Schematic summarizing studies of postmenopausal women with a history of preeclampsia. During normal pregnancy, blood pressure is reduced (green). However, during a preeclamptic pregnancy, blood pressure is elevated (red) after 20-week gestation, continues to be elevated throughout the pregnancy, and decreases back to prepregnancy levels after delivery. Aging and menopause increase risk for hypertension in women, and a history of preeclampsia augments hypertension risk. Postmenopausal women with a history of preeclampsia had impairments in neurovascular control that were associated with increased vascular activation compared with women who had a history of a normotensive pregnancy [68••]. Gray matter atrophy in the occipital lobes were also observed in women with a history of preeclampsia and current controlled hypertension [73••] as were increased volume of white matter lesions as early as < 10 years postpartum [71]. Brain structural changes were consistent with deficits in visual memory, as well as a trend for increased incidence of clinical cognitive impairment diagnosis with a more diffuse range (i.e., multiple domains) in cognitive impairment [74••]. We hypothesize that reduced neurovascular control is a potential mechanism by which a history of a preeclamptic pregnancy may augment risk for cognitive decline later in life. It is unclear at this time whether, when, and how reduced neurovascular control may progress in women with a history of a normotensive pregnancy as they age. It was also unclear whether the changes in the occipital cortex observed in hypertensive women with a history of preeclampsia are specific to that complication of pregnancy. This figure was created in part with modified Servier Medical Art templates, which are licensed under a Creative Commons Attribution 3.0 Unported License: https://smart.servier.com

In a prospective study of a carefully defined cohort of postmenopausal women with a history of preeclampsia and parity-matched women with a history of a normotensive pregnancy who had otherwise low cardiovascular risk, having a history of preeclampsia had an impact on neurovascular control and vascular anatomy characterized by increased coronary artery calcification and increased carotid intima-media thickness. Structural changes to the central arteries were reflected by increased central blood pressures that were associated with reduced cognitive testing scores only in women with a history of preeclampsia. In these postmenopausal women with a history of preeclampsia and current hypertension, CVR was reduced contributing perhaps to reduced total gray matter volumes and regional gray matter volume (Fig. 1). Furthermore, having current hypertension amplified gray matter atrophy in the occipital lobes that was consistent with deficits in visual memory in women with a history of preeclampsia. There was trend for women with history of preeclampsia to have increased incidence of clinical cognitive impairment diagnosis with a more diffuse range (i.e., multiple domains) in cognitive impairment compared with women who had a history of a normotensive pregnancy (Fig. 1).

These data add to the mounting evidence suggesting the detrimental impact of hypertension, especially during early and midlife, on cognition later in life. In 2017, the American Heart Association revised the guidelines for the diagnosis and treatment of hypertension by lowering the threshold for both systolic and diastolic blood pressures by 10 mmHg [79]. Given that some changes in the brain structure in women, related to development of WMH, can occur with blood pressures < 140/90 mmHg [52], earlier interventions for blood pressure control could reduce these unfavorable changes in brain structure. Treating hypertension at the earliest stages is critical, as little is known about how duration of untreated hypertension or duration of anti-hypertensive treatment can compound the effects on the cerebral blood vessels, neurovascular coupling, and brain structure. As preeclampsia is a hypertensive episode, and a history of preeclampsia elevates cardiovascular risk, monitoring blood pressure in women with histories of preeclampsia after pregnancy and around perimenopause is essential. A simple validated questionnaire to inquire about the pregnancy history of women, if unknown, could be useful in clinical or research environments to help establish this potential risk [80]. In addition, studies and clinical trials aimed at attempting to assess the effect of blood pressure control on risk for Alzheimer’s disease and other dementias should consider how their results are impacted by hormonal changes experienced by women throughout the life span (i.e., pregnancy and menopause) before making generalized conclusions about the effectiveness of the trials [81, 82].

In summary, study of a defined cohort of postmenopausal women who had a history of either a preeclamptic or normotensive pregnancy has provided insights into the associations of hypertension, central artery structure and functional changes, cerebrovascular function, brain structure, and cognition. These results can generate hypotheses for other longitudinal studies that will help to identify mechanisms by which changes in the hormonal milieu contribute to cerebrovascular aging, brain structure, and ultimately cognition.
